# Creative arts in psychotherapy for traumatized children in South Africa: An evaluation study

**DOI:** 10.1371/journal.pone.0210857

**Published:** 2019-02-13

**Authors:** Nadine van Westrhenen, Elzette Fritz, Adri Vermeer, Paul Boelen, Rolf Kleber

**Affiliations:** 1 Department of Clinical Psychology, Utrecht University, Utrecht, The Netherlands; 2 Athena Institute, Vrije Universiteit Amsterdam, Amsterdam, The Netherlands; 3 Department of Educational Psychology, University of Johannesburg, Johannesburg, South Africa; 4 Department of Education and Pedagogics, Utrecht University, Utrecht, The Netherlands; 5 Arq Psychotrauma Expert Group, Diemen, The Netherlands; University of California, San Francisco, UNITED STATES

## Abstract

**Aim:**

To evaluate the feasibility and effect of a 10-session creative arts in psychotherapy group programme on posttraumatic stress symptoms, behavioural problems, and posttraumatic growth, in children who experienced a traumatic event.

**Design:**

A multicentre non-randomized controlled trial with a treatment and a control condition conducted in South Africa (4 sites).

**Methods:**

125 children aged 7 to 13 years were assigned either to the treatment condition receiving creative arts in psychotherapy or a control condition with a low-level supportive programme without treatment. Attrition rates were 63.4% and in total 47 children completed the programme and questionnaires assessing posttraumatic stress, posttraumatic growth and behaviour problems both at baseline and follow-up; 23 in the treatment group and 24 in the control group. Adjusted mean differences were analysed using ANCOVA with bootstrapping.

**Results:**

Results showed that both hyperarousal symptoms (*d* = 0.61) and avoidance symptoms (*d* = 0.41) decreased more in the treatment group compared to the control group. There was no significant effect of the intervention found for reported levels of behavioural problems and posttraumatic growth.

**Conclusion:**

In spite of severe challenges implementing and executing this pioneering study in underprivileged areas of South Africa, support was found for creative arts in psychotherapy reducing hyperarousal and avoidance symptoms, but not for other symptoms. Valuable lessons were learned on feasibility of implementing this intervention in a developing context.

## Introduction

### Trauma exposure

For a country not at war, South Africa is a place with extreme high rates of traumatic exposure, with one of the highest rates of interpersonal violence and domestic abuse in the world [1]. Violence against children is especially pervasive, from severe beatings to sexual violence and rape [1]. Exposure has been reported as high as 98.9% for community violence [2] and 54.2% for sexual abuse [3]. The high exposure to interpersonal violence increases vulnerability to mental disorders such as posttraumatic stress disorder (PTSD) [4,5]. High levels of PTSD have been reported amongst poor urban children in South Africa and prevalence estimations have been published of 22.2% and 23.6% [5, 6]. Apart from PTSD, children exposed to interpersonal violence are more likely to experience a wide range of adverse psychological problems, such as depression, suicidality, and substance abuse [5,7] and externalizing behaviour problems such as violent and anti-social behaviour [8].

Apart from negative psychological consequences of abuse and neglect, positive change may also result from traumatic exposure, called posttraumatic growth (PTG) [9]. PTG generally includes five domains; relating to others, personal strength, appreciation of life, spiritual change, and new possibilities [10]. PTG has mostly been studied in adults and there is a growing body of literature describing the phenomenon of PTG in children and adolescents [11, 12].

### Child trauma treatment

Different types of interventions have been proven to be effective for improving mental health in traumatized children. Particularly cognitive behaviour therapy (CBT) has emerged as one of the most effective and widely used treatments [13, 14]. More evidence, however, is required in order to establish the effectiveness of different therapies in the longer term, and for comparability of different types of therapy, such as CBT, play therapy, eye movement desensitization and reprocessing (EMDR), art therapy, and psychodynamic therapy [13]. Moreover, since most studies have been conducted in high-income countries, evidence is required for the applicability of trauma interventions in a low- and middle-income context [15].

Creative arts therapy is a widespread approach in the treatment of child post trauma disorders. It includes different modalities of art, music, dance and drama in combination with other approaches to psychotherapy and counselling [16], such as psychodynamic, cognitive, developmental, systems, and narrative therapy. The use of art therapy practise varies and can be described on a continuum [17], ranging from using arts as an adjunct in verbal psychotherapy to art engagement without verbal analysis, and several gradations in between. Creative arts therapies used as a primary form of therapy requires graduate-level training in one or more modalities. Creative arts is also used by counsellors or other qualitied mental health professionals in facilitating different stages in psychotherapy [18]. When using creative arts in this way as an adjunct, the key is to understand the various treatment goals and to carefully select creative arts activities that can support this process [19]. Mental health professionals can be offered training in applying creative arts activities in psychotherapy, but at all times it is important to avoid challenging ethical boundaries by going beyond what someone was trained to do.

Research with children found that the use of art can facilitate exposure to traumatic cues in a non-threatening manner, allowing for desensitization of anxiety, articulation of affective states [20], and more detailed and emotional narratives [21]. Also, arts-based methods can assist children in developing coping skills, self-awareness and aspects of self-esteem [22], with the creative process providing a containing space in the relationship with the therapist [23]. Facilitating (creative) therapies for traumatized children in a group setting can have additional benefits, because group members can facilitate trust and disclosure, providing an opportunity for children to realize they are not alone in their problems, and finding peer-support [24, 25]. Creative arts activities can reinforce these group benefits by serving as a medium for communication, for instance dance/movement can help establish a sense of connection and understanding between people [26], and music provides a medium to communicate and build relations [27].

Although the possibilities of creative arts therapy appear promising, there is very little research available on the efficacy of such therapies for children after trauma [28, 29]. Only a few studies have explored the effects of creative therapy for children on successfully reducing posttraumatic stress symptoms [30, 31] and behavioural difficulties [32]. In the South African context, to our knowledge, only one group art therapy intervention for sexually abused girls from 8 to 11 years old has been evaluated [33]. This study showed positive results regarding anxiety, depression and traumatic stress symptoms, but no effect of the intervention was found on levels of self-esteem. Apart from this study, methodologically sound studies focusing on the effects of creative arts therapy on specific outcome measures are scarce [34].

### Study purpose

The present study aims to assess the possible influence of a creative arts in group psychotherapy programme for traumatized children in South Africa on posttraumatic stress symptoms, behaviour problems and PTG. We performed a non-randomized controlled trial comparing creative arts in psychotherapy with a low-level supportive programme including no art and/or psychotherapeutic interventions and hypothesized that a creative arts in psychotherapy programme (CAP) is more efficacious compared to the control condition in 1) reducing posttraumatic stress symptoms, 2) reducing behavioural problems, and 3) increasing PTG, in children who experienced one or more traumatic events.

## Methods

### Design

This was a multicentre non-randomized controlled trial conducted in South Africa (4 sites) with two conditions, and including 3 measurements at baseline and follow-up.

### Sampling

The study took place at four branches of a child abuse clinic in Johannesburg, South Africa, from January 2014 to June 2016. Children attending this clinic come from various communities (mostly townships and informal settlements) in and around Johannesburg, represent different racial groups (although primarily Black, also Coloured, Indian and White families attend) and speaking different home languages (South Africa has 11 official languages).

125 participants were selected for this study from all children that came for intake at the trauma clinic, based on the following inclusion criteria: (1) experienced one or multiple events of trauma or abuse between three months and twelve months ago; (2) developmental age between 7 and 13 years at the time of enrolment; (3) can speak English in order to communicate with all social workers and peers in therapy. Exclusion criteria were (1) mental retardation, autistic disorder, and blindness, (2) already had any form of previous trauma treatment. Selection for the different conditions was done non-random due to practical considerations relating to the limited availability of participants. One group of social workers who were trained in the CAP programme invited all children meeting inclusion criteria during their intake at the clinic to participate in the therapy (n = 74). Other social workers in the clinic continued to refer children to treatment as usual, starting with a non-therapeutic court preparation programme whilst awaiting availability for individual play therapy. All children attending this court preparation programme at the clinic meeting inclusion criteria were also invited to participate in the control condition (n = 51).

### Outcome measures

#### Posttraumatic stress symptoms

Posttraumatic stress symptoms were measured by the Child PTSD Checklist (C-PTSD-C) [35]. This self-report measure is a 28-item checklist that rates DSM-IV-TR characterized PTSD symptoms in the past month. The scale uses a 4-point Likert scale, ranging from ‘not at all’ (scored 0) to ‘all the time’ (scored 3), with higher scores indicating more severe PTSD symptoms. The C-PTSD-C has three subscales: Hyperarousal, avoidance, and reexperiencing. Psychometric properties have been published in the South African context [36], and the instrument was found to be a reliable and valid measure of PTSD symptoms. Internal consistency for the scale in the current sample was good between α = .78 (baseline) and α = .90 (follow-up).

#### Behaviour problems

Behaviour problems were reported by the parents or a close relative on the Child Behaviour Checklist (CBCL) [37]. This checklist consists of 120 items, assessing emotional and behavioural problems, rated on a 3-point scale ranging from ‘not true’ (scored 0) to ‘very true or often true’ (scored 2). The CBCL has three main scales, internalizing, externalizing and total problems, as well as eight sub-scales comprising specific behaviour domains. Research using the CBCL has demonstrated its sound reliability and validity across multiple cultural settings [38]. Internal consistency in the current sample was excellent (baseline α = .96, follow-up α = .96).

#### Posttraumatic growth

PTG was measured with the self-report Posttraumatic Growth Inventory for Children- Revised (PTGI-C-R) [10]. The instrument has 10 items using a four-point Likert scale ranging from no change (scored 0) to a lot (scored 3). Research findings demonstrate validity and reliability of the revised scale [10]. Previous studies measuring PTG in low-income settings, although rare, demonstrate positive results [39, 40]. Internal consistency for the full scale in the current sample was found between α = .70 (baseline) and α = .76 (follow-up).

### Conditions

#### Treatment condition

Children in the treatment condition attended the Creative Arts in Psychotherapy (CAP) intervention [41]. CAP was a structured programme of ten 90-minute sessions, specifically developed for traumatized children in the age between 8 and 12 years. The sessions were facilitated once a week in closed groups of six to eight participants by local social workers trained by creative arts therapists to conduct arts-based activities in psychotherapeutic practice, and different multimodal activities incorporating visual art, movement, dance, drama, music, and storytelling were used to work towards specific session goals. The programme outline was based on the three phases of the treatment model for severely traumatized individuals [42]. The first three sessions focused on establishing safety, and activities included for instance having children mirror each other’s dancing to increase connection between group members [43] and reading and discussing a children’s story of ‘a terrible thing happened’ [44]. Sessions four to six aimed to facilitate expression of emotions associated with the trauma story and practise emotion regulation by for instance decorating masks to express and distinguish between feelings from the inside and what others see from the outside. Lastly, the final four sessions focused on strengthening coping skills by for instance writing and socio-drama activities incorporating the hero’s journey [45] and making music together using drums and other music instruments. Overall, the intervention aimed to improve identification and communication of emotions, interpersonal skills and intrapersonal connectivity and resilience to cope with future crisis, increase PTG, and reduce posttraumatic stress symptoms.

#### Control condition

The control group did not attend any therapy, but a so called ‘court preparation and support programme’. This non-therapeutic programme focused on providing children and parents skills, emotional support and legal knowledge in preparation for their appearance in court. This programme was part of the treatment as usual in the clinic, after which the children were offered the opportunity to attend individual play therapy. The programme was facilitated by social workers during monthly three-hour open group sessions, where children could join as long as necessary while the court case preparations were still ongoing. The children in this control condition attended about 3 sessions (over a 2-month period) during the time this study took place. The sessions were solely focused on the court process, and not on any psychosocial impact of the trauma on the client’s personal life.

### Procedure

To detect a change in the dependent variables between the two conditions, with a two-sided 5% significance level, medium effect size and a power of 80%, a sample size of 64 per group was necessary [46]. Baseline questionnaires were administered on paper prior to the first session of the CAP programme (treatment condition) and during the first monthly court preparation session the child was attending (control condition). Follow-up questionnaires were subsequently administered after the final session of the creative therapy programme, and during another court preparation session on average two months after baseline measurements. In a number of instances there were reading challenges and the questionnaires were administered verbally, and individual appointments were arranged to administer questionnaires if the parents and children were not available for the group sessions in which the questionnaires were administered. For those children who did not complete the full programme, post-measurement questionnaires were still administered, adhering to the intention-to-treat approach.

### Ethical approval

Permission for this study was obtained from the Faculty of Humanities Academic Ethics Committee of the University of Johannesburg. Informed consent and informed assent was obtained from the children and their parents or primary caregiver prior to participation in this study. Participation was volunteer and confidential. Children in the control group were offered the option to attend therapy at the clinic after participating in the court preparation and support programme, and children participating in the CAP could also afterwards join the court preparation and support programme.

### Data analysis

Analyses were conducted using IBM SPSS statistics 22. Missing data on item level were replaced using multiple imputation. The multiple imputations appeared similar and comparable, and therefore one imputation was selected to allow for subsequent analysis including bootstrapping. Baseline analyses were performed using bivariate analysis, exploring differences on treatment condition, gender, race, type of trauma and baseline measures of PTSD, PTG and behavioural problems. To explore the treatment effect, the mean difference score between baseline and follow-up measurements was compared between the different test conditions (treatment vs control) using ANCOVA with ethnicity and type of abuse as covariates. Considering the small sample and non-normal distribution of data, bootstrapping techniques were applied.

## Results

### Feasibility

From the 125 children initially referred to the programme, 62.4% dropped out in both the treatment and control condition. Of the four different branches, treatment groups of two branches had to be terminated prematurely due to high dropout rates. One group was facilitated in a place of safety, and turnover rates of children in this place was very high. For the other branch, reasons for drop out were mostly related to accessibility. Parents reported traveling up to two hours from home to the clinic and struggled to afford the transport costs or were not able to take time off from work to bring the children [47]. The other 2 branches had relatively sufficient turnout over a 2-year period. These branches were more centrally located within a specific community. For the control group, monthly turnout was inconsistent. Some parents and children would show up every month, but most children only attended once or twice, and then disappeared off the radar.

Despite the challenges with attrition and inconsistent turnout, a total of 23 children completed the CAP programme. These children received an average of 5.52 (SD = 3.20) sessions during a period of 10 weeks. This equals 8.28 hours of therapy. Six children in the treatment group who received CAP only attended one or two sessions out of the prescribed ten.

### Participants flow

**Fig 1:**
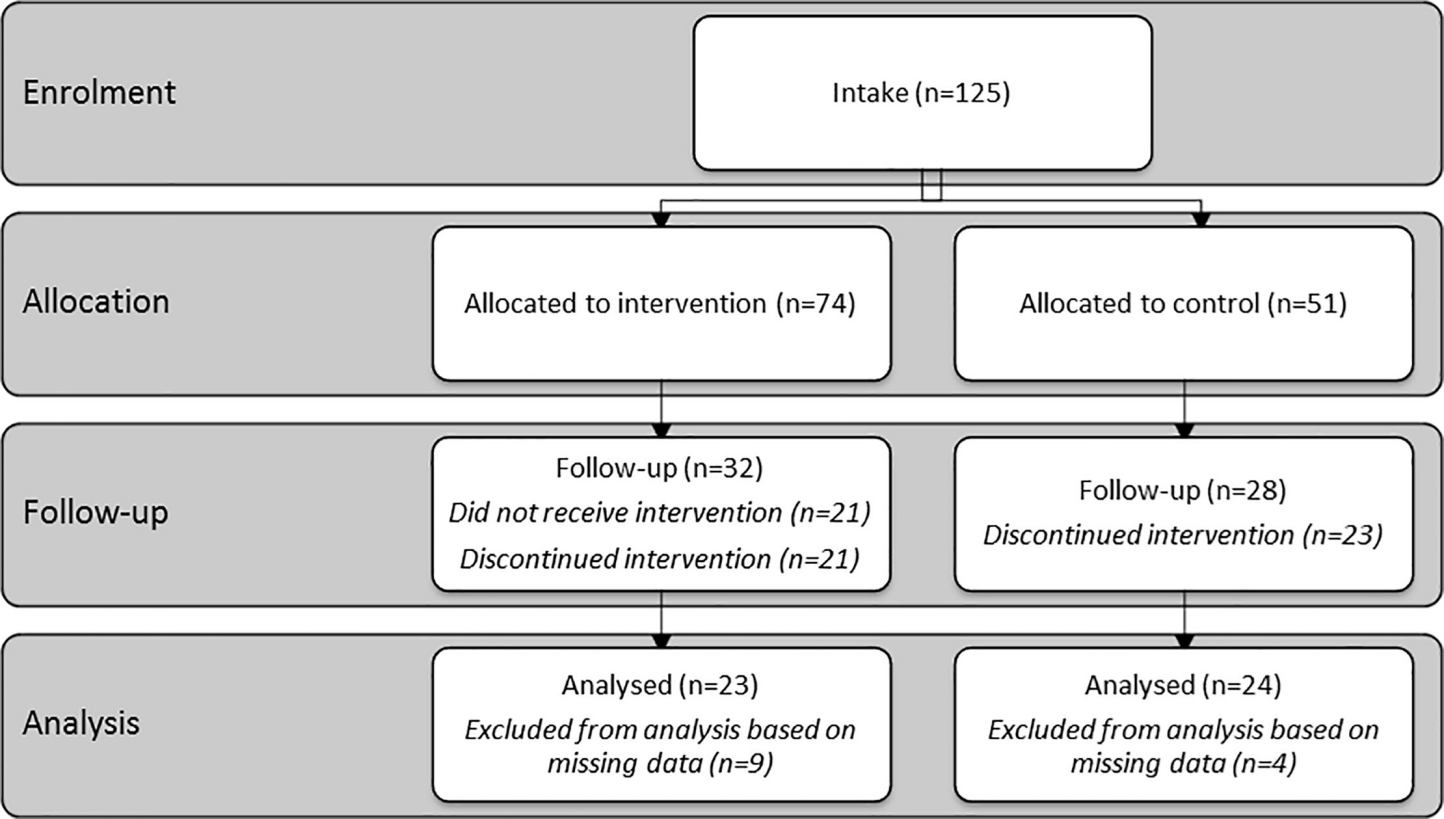
Flow diagram of progress through the phases of the experimental trial of two groups.

In total 125 children participated with baseline measurements in the study. Subsequently, social workers referred 74 participants to the treatment condition, and 51 participants to the control condition. For the treatment condition, after participant dropout (n = 42) and exclusion of participants who attended the intervention but did not complete the post-measurements (n = 9), a total of 23 participants were included for the analysis of the C-PTSD-C and the PTGI-C-R and a total of 18 participants were included for the analysis of the CBCL. The CBCL was completed by the parents and often the children travelled alone or with a sibling to the clinic, making it hard to get hold of the parents for completion of questionnaires. For the control condition, after dropout (n = 23) and exclusion of those who completed only one out of the three post-measurements (n = 4), analyses were conducted with a sample of 24 participants for the C-PTSD-C and the PTGI-C-R, and 19 participants for the CBCL. A summary of the participants’ flow through the different project stages is provided (Fig 1).

The final sample of 47 participants for analysis of the C-PTSD-C and the PTGI-C-R consisted of 23 children in the treatment group, 3 boys and 20 girls, aged between 7 and 13 (M = 10.14, SD = 1.92). The control group consisted of 24 children, 8 boys and 16 girls, aged between 8 and 13 (M = 10.50, SD = 1.32). The final sample of 37 participants for analysis of the CBCL consisted of 18 children in the treatment group, 2 boys and 16 girls, aged between 7 and 13 (M = 9.93, SD = 1.94). The control group consisted of 19 children, 6 boys and 13 girls, aged between 8 and 13 (M = 10.30, SD = 1.38). The majority of children in the study had experienced sexual abuse, five children experienced physical abuse (Table 1).

**Table 1:** 

	Sample for PTSD & PTG		Sample for CBCL	
	Treatment (n = 23)	Control (n = 24)	Treatment (n = 18)	Control (n = 19)
Age (years)	10.14 (1.92)	10.50 (1.32)	9.93 (1.94)	10.30 (1.38)
Gender (female)	20 (87.0%)	16 (66.7%)	16 (88.9%)	13 (68.4%)
Ethnicity:				
African	22 (95.7%)	16 (66.7%)	18 (100%)	12 (63.2%)
Asian	-	2 (8.3%)	-	2 (10.5%)
Coloured	1 (4.3%)	2 (8.3%)	-	1 (5.3%)
White	-	4 (16.7%)	-	4 (21.1%)
Type of trauma:				
Sexual abuse	23 (100%)	17 (70.8%)	18 (100%)	14 (73.7%)
Physical abuse	-	5 (20.8%)	-	3 (15.8%)
Other	-	2 (8.3%)	-	2 (10.5%)

Note. PTSD = posttraumatic stress disorder, PTG = posttraumatic growth, CBCL = child behaviour checklist.

### Baseline data

The treatment group (M = 26.78, SD = 11.48) and control group (M = 33.99, SD = 11.57) differed significantly on PTSD symptoms at baseline (t(45) = -2.143, p < .05*)*. Fisher’s exact test for the sample of 47 participants did show that there were significantly more black children in the treatment group (95.7%) compared to the control group (66.7%; p < .05), and there were also more children that were sexually abused in the treatment group (100%) compared to the control group (70.8%; p < .01). Also for the sample of 37 participants, there were more black children in the treatment group (100%) compared to the control group (63.2%; p < .01), and more children had been sexually abused in the treatment group (100%) compared to the control group (73.7%, p < .05). Other variables tested did not differ significantly across conditions.

### Evaluation of outcomes

Controlling for the effect of ethnicity and type of abuse in an ANCOVA, bootstrapped adjusted mean differences showed that hyperarousal symptoms significantly decreased in the treatment condition between baseline and follow-up (from M = 10.39 to M = 6.77, *d* = 0.61), where it slightly increased for the control group (from M = 6.73 to M = 7.46, *d* = -0.15; adjusted mean difference = 4.36, 95% CI 0.36, 8.69). Moreover, avoidance symptoms decreased significantly more for the treatment condition (from M = 13.48 to M = 11.13, *d* = 0.41) compared to the control condition (from M = 11.05 to M = 10.99, *d* = 0.01; adjusted mean difference = 4.11, 95% CI 0.03, 8.42), yet the effect size was small. Overall PTSD symptoms, as well as reexperiencing symptoms also decreased in the treatment condition, but not significantly more than in the control condition, see Table 2.

**Table 2:** Summary results treatment and control group.

	Treatment		Control		Adjusted mean difference** (95% CI)
Scale (range)	Baseline (mean (SD))	Follow-up (mean (SD))	Baseline (mean (SD))	Follow-up (mean (SD))	
PTSD symptoms	n = 23		n = 24		
Total (0–84)	33.99 (11.57)	27.06 (18.18)*	26.78 (11.48)	26.84 (12.68)	9.40 (-0.18, 20.01)
Avoidance (0–30)	13.48 (4.78)	11.13 (6.63)	11.05 (5.15)	10.99 (4.54)	4.11 (0.03, 8.42)***
Reexperiencing (0–27)	9.43 (5.25)	8.64 (6.42)	8.75 (3.96)	7.83 (4.59)	0.33 (-3.03, 3.60)
Hyperarousal (0–24)	10.39 (4.96)	6.77 (6.72)*	6.73 (4.20)	7.46 (5.26)	4.36 (0.36, 8.69)***
Behaviour problems	n = 18		n = 19		
Total (0–240)	62.91 (35.97)	48.98 (33.66)	71.35 (37.01)	51.46 (28.12)*	-13.90 (-53.28, 20.88)
Internalizing (0–78)	18.81 (11.76)	14.59 (11.14)	21.08 (10.45)	14.19 (7.60)*	-5.80 (-16.65, 3.91)
Externalizing (0–70)	16.68 (10.09)	14.05 (9.75)	20.22 (13.45)	16.15 (11.62)	-2.12 (-14.23, 8.20)
Posttraumatic growth	n = 23		n = 24		
Total (0–30)	22.34 (5.29)	23.99 (4.42)	19.75 (5.25)	23.44 (5.01)*	1.63 (-2.70, 6.08)

*Difference within condition between baseline and follow-up significant at p < .05

**Mean difference score between conditions adjusted for ethnicity and type of abuse based on 1000 bootstrap samples; PTSD = posttraumatic stress disorder.

*** Difference in changes between treatment and control condition significant at p < .05

**Fig 2:**
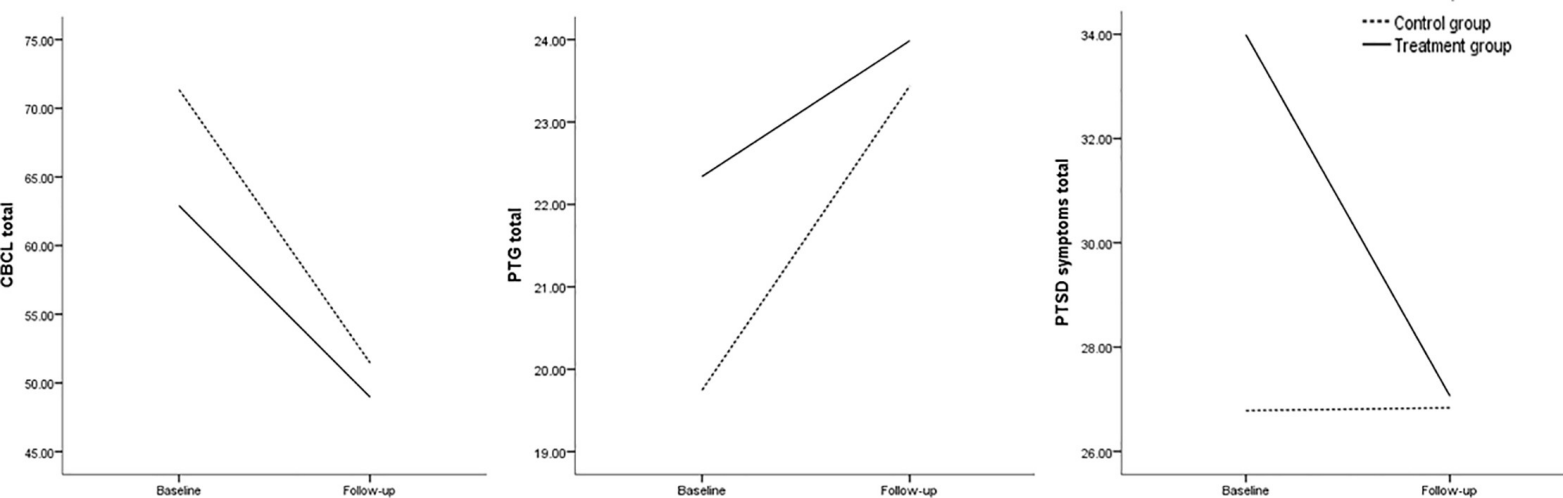
Posttraumatic stress symptoms, behaviour problems and posttraumatic growth scores of the treatment and control groups at baseline and follow-up. The scores are mean total scores.

Behaviour problems also showed a decrease over time in both the treatment condition (from M = 62.91 to M = 48.98, *d* = 0.40) and the control condition (from M = 71.35 to M = 51.46, *d* = 0.61), and internalizing behaviour decreased more than externalising behaviour, but these changes were not statistically significant when compared between conditions. Lastly, PTG increased in both the treatment condition (from M = 22.34 to M = 23.99, *d* = 0.34) and the control condition (from M = 19.75 to M = 23.44, *d* = 0.72), but there was no significant difference in this increase between conditions (Table 2 and Fig 2).

## Discussion

Evaluation of the CAP programme showed that compared to the control condition, hyperarousal symptoms decreased significantly more during CAP with a medium effect size for the pre-treatment to follow-up change scores. Avoidance symptoms also decreased more during CAP than in the control condition, but the effect size was small. No support was found for our hypothesis that the CAP programme is more efficacious than a low-level supportive programme in reducing reexperiencing symptoms, behaviour problems and increasing PTG.

In addition to these quantitative findings, the social workers were positive about the intervention. They observed the children moving from the point of being victim to survivors, the children were smiling and interacting more and demonstrating more confidence. The social workers noticed that the creative activities provided the children a platform to express their emotions and show their talents and the children found empathy from their fellow group members and felt supported. Other positive feedback we received was that children were telling us that they were sleeping better now, and some parents told us that their children became more playful and showed less resistance at home.

Below we will discuss the outcomes of the intervention and lessons learned on feasibility by reflecting on barriers in recruitment and retention and discussing methodological limitations.

### Therapeutic outcomes

Previous studies highlighted the positive effects of creative arts therapy specifically on reducing psychological stress [48], having a soothing capacity [16, 49] and establishing a sense of safety [50]. This may in turn have facilitated decreased hyperarousal symptoms and helped regain or develop healthy emotion regulation after experiencing severe stress. The positive effect of group therapy and activities facilitating emotional expression and working through the traumatic experience may have contributed to reduced avoidance symptoms.

The creative arts in psychotherapy programme (CAP) did not diminish reexperiencing PTSD symptoms, behaviour problems and PTG as successfully. It could be that the therapeutic activities in the treatment protocol did not address all these different outcome measures as purposefully as intended, or perhaps the creative arts activities facilitated by trained social workers were inferior to the delivery of creative arts therapy by trained and credentialed creative arts therapists. Possibly, the lack of a direct trauma-exposure component in the treatment may also have affected the outcomes. Currently, there is a debate whether directly facilitating re-exposure in therapy would be more beneficial [51]. On the one hand, it has been found that trauma-focused treatments show higher effect sizes compared to non-trauma-focused treatments [52], yet a recent meta-analysis showed this difference is rather small and not clinically meaningful [53]. Moreover, exposure therapies are also associated with an early and high dropout and patients having remaining symptoms [54, 55].

Moreover, not all these interventions have been shown to be effective in a context of ongoing adversity such as chronic poverty, community violence and war [15, 56, 57]. Therefore, another explanation for our programme evaluation results may be that the circumstances of ongoing adversity are impeding the potential therapeutic benefits of the intervention. We also noticed that most trauma treatment studies in a developing context have focused solely on PTSD and internalizing symptoms as outcome measures [15]. Perhaps other outcomes such as externalizing responses, but also resilience, self-confidence, and social support could be more relevant in a setting of poverty, hardships and crime and should be an essential focus in future studies.

Lastly, a lack of significant therapeutic outcomes for reexperiencing symptoms, behaviour problems and PTG could also be attributed to improvements in the control group. Although the control condition was considered non-therapeutic, it could be that the programme did provide the children with certain coping skills, for instance on how to deal effectively with their court appearance and the anxiety around this appearance. In this way, both the treatment and control group could have addressed self-regulation skills, which is considered an important mechanism for processing the sensory experience of trauma in the body [58]. The presence of other children in the control condition who all went through a similar traumatic event and the help of social workers in the programme could also have increased their sense of social support and security. This could have resulted in unexpected therapeutic effects in the control condition, decreasing chances of detecting significant differences between the study conditions.

### Recruitment and retention

Despite the very high rates of abuse and trauma exposure in South Africa and their negative psychological consequences [1], few children enrolled and completed the creative therapy programme. Unfortunately, these difficulties in reaching patients and high dropout rates of mental health treatment are well-known issues in a low and middle income context [59, 60].

Our implemented intervention programme aimed to explicitly address previously reported structural barriers with availability and accessibility of services by working from a decentralized location in and around the townships at four different sites, building capacity of skilled health care workers through training and supervising social workers, and offering the therapy free of charge [15, 61]. For two branches this was quite successful, but for two other branches it was still a challenge to reach the target population. In future, we may look to collaborate with schools or churches to improve accessibility and aim to shorten the 10-week programme to decrease travel time and possibly reduce dropout rates.

Moreover, acceptability of the treatment may have remained a problem amongst the target population. Many people in South Africa use traditional explanatory models of health, referring to spiritual causes of ill health such as ancestors, for which they seek the help of a traditional healer instead of a medical or psychological professional [62]. Although the creative arts in psychotherapy tried addressing the gap between the western and more traditional practises, by incorporating creative expressions that are used in traditional rituals such as masks, dancing and drumming, the concept of therapy may still have been too foreign for the community and more education is needed in this area. Moreover, in a context of extreme poverty, priorities could have been with finding food and shelter rather than seeking help for mental health problems.

The problem of recruitment and retention, and treatment accessibility and acceptability in crime-stricken and underprivileged settings such as in South African townships and informal settlements deserves even more serious consideration than presumed. It would be effective in these problematic socio-economic circumstances to combine therapy interventions with programmes explicitly focusing on mental health education providing an intrinsic motivation for therapy attendance [59]. Such an approach would also fit into the emphasis on social connectivity in trauma care as suggested by several authors on global health [63].

### Methodological limitations

Due to several practical challenges in the research project, the study had to be implemented with more flexibility and therefore less rigor than initially intended. This resulted in inconsistencies in data collection, decreasing the value of evidence of this study. Clearly, the small sample size and insufficient possibilities for randomization were a substantial limitation in this study. The current results may not accurately reflect the possible full potential of the creative arts in psychotherapy programme as mentioned above.

There were limitations with the sampling in this study. First, only children who could speak English were included in this study. However, in South Africa not everyone can speak English. Furthermore, despite this inclusion criteria we still experienced problems with language barriers as the English of some children and parents was not sufficient and low literacy rates also complicated administration of the questionnaires. In order to address these language barriers, we introduced translations and visualisations and therapy was facilitated by social workers who spoke the home language of the children. Second, at the start of the study there were already differences between the intervention and the control group; for instance the treatment group reported significantly more PTSD symptoms than the control group. Another difference was that 100% of the children in the treatment group were sexually abused, whereas in the control group this was 70.8%. Initially, the treatment group included also children who were physically abused, but they were amongst those who dropped out. Referring to both examples above and knowing that on average children who were sexually abused display more severe symptoms of PTSD, possibly parents of children with more severe PTSD symptoms were more willing to participate in the therapy.

In view of the complexity of the South African setting in which this study was conducted, we recommend the use of mixed methods for future studies in a similar context, incorporating for instance interviews, focus-groups and observational data to add to standardized questionnaires and interviews [64]. In this way, we can enrich the knowledge on how to implement evidence-based treatment for traumatized and abuse children in developing countries more effectively.

### Conclusion

This pioneering study conducted in South Africa investigated the potential effects of a creative arts in psychotherapy intervention programme for traumatized children. Although severe challenges implementing and executing the study limited the power of this evaluation study, results also show positive findings. We hope our insights will inspire more work in this area. Considering the high need for evidence-based trauma care for children in low income countries, we recommend more studies to be conducted on the efficacy of creative arts in psychotherapy and the effects of trauma-intervention studies.

## Supporting information

S1 File CBCL datafile.dat. (DAT)

S2 File PTSD & PTG datafile.dat. (DAT)
